# Smallholder Farmers Contribution to Food Production in Nigeria

**DOI:** 10.3389/fnut.2022.916678

**Published:** 2022-07-28

**Authors:** Jeffrey Chiwuikem Chiaka, Lin Zhen, Hu Yunfeng, Yu Xiao, Fabien Muhirwa, Tingting Lang

**Affiliations:** ^1^Key Laboratory for Resources Use and Environmental Remediation, Institute of Geographic Sciences and Natural Resources Research, Chinese Academy of Sciences, Beijing, China; ^2^College of Resources and Environment, University of Chinese Academy of Sciences, Beijing, China; ^3^Anambra-Imo River Basin Development Authority, Owerri, Nigeria

**Keywords:** staple foods, smallholder farmers, food production, satiety, population density, per capita food supply, Nigeria

## Abstract

Several studies have shown that smallholder farmers produce most of the food in low-income and developing countries and form the backbone of the country’s food supply. This study examines the extent these smallholder farmers in Nigeria can put the country on the path to self-sufficiency and ensure satiety for household food consumption through their local production. The study also examines food production and their resulting yield based on crop production and harvested area, as well as the percentage of crops produced for food or other purposes. The results show that production of rice, sorghum, soybean, cassava, and yam is low; and their corresponding yields are declining, with the exception of maize, although the harvested area increased from 2015 to 2018. As it is, the findings are a clear indication of inadequate per capita food supply due to low food production, especially for cereals. The study suggests closing the yield gap specifically for cereals, limiting post-harvest losses, and finding a sustainable balance between the uses of major food crops for animal feed to reduce pressure on land resource use. The different states production performance requires special attention to harness the agricultural potential of each geopolitical zone. Lastly, dry-season cultivation should be encouraged through irrigation to enable harvesting two-times in a year. The study offers useful approaches to assess the contribution of local farmers to the food supply of a growing population and provides suggestions for the government, stakeholders, and the international community willing to collaborate and invest in the agricultural sector.

## Highlights

- The country has a low cereal production.
- Food waste due to post-harvest loss remained constant for the major food crops from 2015 to 2018.
- Despite the increase in harvested area, crop yields were on a decline from 2015 to 2018.
- Most densely populated states have low to medium food production and are likely to be vulnerable to food accessibility.

## Introduction

There is an increasing call for sustainable food production to meet growing demands in the world, especially in countries faced with the challenge of hunger, poverty, insufficient food consumption, and malnutrition in Africa (1–4). An estimated 75% of the world’s population relies on agriculture for food and income (5, 6), which is the most important factor driving the expansion of cropland worldwide (7–10). Furthermore, studies show that 62% of the world’s arable land is used for cropping, 3% for bioenergy, and 35% for animal feed, i.e., meat production (11). For instance, it is estimated that 40% of arable land in North America and Europe is used for food production, while more than 80% of arable land in Africa and Asia is used for food production (11).

The United Nation Conference on Trade and Development (UNCTAD) suggests that addressing food security entails recognizing smallholder farmers for their role to achieve hunger and poverty reduction through their active participation in food production (12). The report also recognizes regional differences in farm sizes cultivated by smallholder farmers. For instance, in Latin America, an average farm size is 20 ha, and Brazil has 50 ha, while in Asia and sub-Saharan Africa, the farm sizes are smaller. For brevity, in Bangladesh, an average farmer has a farm size of 0.5 ha, while China, India, and Nigeria have less than 2 ha (12). In the UNCTAD report, smallholder farmers are those who cultivate 2 ha or less of land and are characterized by the type of crops grown and the labor utilization, as well as low access to financial credit and markets.

Consequently, smallholder farms are said to be more technically efficient in terms of labor which comes from the household to maximize production and reduce cost, while large farm sizes are labor intensive and, if not properly supervised, lead to low food production (13). In addition, the ability of smallholder farmers to mitigate environmental particularities in sub-Saharan Africa is by the adoption of local methods, using native knowledge and experience past down from generations to mitigate perceived harsh environmental conditions for farming activities (14). Moreover, from a biodiversity standpoint, these smallholding farmers are seen as biodiversity friendly as most of them cannot afford fertilizers and are into mixed farming, and make use of livestock manure on their farms (15). This is compared to large farm holders who use intensified means, such as heavy farm machineries, fertilizers, and pesticides on their farms, which influences land degradation (16).

In the area of production, a study opines that smallholder farms produce more than large farms (13, 16), which is described as the inverse relationship between farm size and productivity. The influence of farm size, productivity, and crop allocation has been documented in various studies in sub-Saharan Africa. Studies in Kenya and Nigeria opined that the inverse relationship phenomenon exists in farm sizes and their productivity as smallholder farmers decide which crops will give them the best harvest and income with minimal inputs (13, 17). In terms of crop allocation, farmers in Malawi under the Farm Input Subsidy Program (FISP) instituted by the government led them to concentrate on few crops and not to diversify their crop productions (18). This is also because smallholder farmers tend to produce more when they receive the necessary external support. Nevertheless, experts were more concerned that the FISP project will not achieve the purpose of nutrition availability and has consequences on soil fertility in the long run (18). However, similar programs are widespread in sub-Saharan Africa, such as the MicroVeg project in Nigeria and the Republic of Benin, on vegetable production and fertilizer use for food security and economic empowerment of rural farming households funded by the Canadian International Food Security Research Fund (CIFSRF). This project benefited participating farmers by improving household food security (19). Hence, mitigating hunger challenges in developing countries is about food satiety and the study examines smallholder farmers contribution to food production and per capita supply in Nigeria.

Few studies have been able to elucidate smallholder farmers food production due to the paucity of data to measure their input in the food system considering policy and programs geared toward improving food production (20), especially in developing countries. This study evaluates smallholder farmer’s contribution to the Nigerian food system, and the objectives of the study are to determine what percentage of the crops produced used for food or other purposes in Nigeria and the second objective is to determine whether the farmers are already able to put Nigeria on the path of self-sufficiency and satiety with their local production.

## Materials and Methods

### Brief Introduction of the Study Area

Nigeria’s estimated population as of 2018 was approximately 196 million (21), with its economy becoming the largest in Africa as of 2014 (22) and blessed with rich natural vegetation and landmass spanning over an area of 924,000 km^2^. Nigeria is a multicultural country with 36 states divided into six geopolitical zones namely: North East, North West, North Central, South East, South South, and South West (Table 1). It is estimated that about 75% of Nigeria’s total land area amounting to about 68 million hectares has agricultural use potential, while about 33 million hectares are actually cultivated. In addition, of the estimated 3.14 million hectares of irrigable land, only about 220,000 ha or 7% is utilized (5). The average farm size for subsistence farmers in Nigeria ranges from 1 to 3 ha, with the North having more farm sizes than the South (22). Furthermore, agriculture employs about two-thirds of Nigeria’s labor force (5). The rainfall pattern across the country decreases from the South to the North. The difference in the mean annual rainfall across the six geopolitical zones is as follows: North Central (269.938 mm), North East (300.794 mm), North West (225.395 mm), South East (442.360 mm), South South (737.836 mm), and South West (323.634 mm) (23).

**TABLE 1:** States in the six geopolitical zones of Nigeria.

S/N	North Central	North East	North West	South East	South South	South West
1	Benue	Adamawa	Jigawa	Abia	Akwa Ibom	Ekiti
2	Kogi	Bauchi	Kaduna	Anambra	Bayelsa	Lagos
3	Kwara	Borno	Kano	Ebonyi	Cross River	Ogun
4	Nasarawa	Gombe	Katsina	Enugu	Rivers	Ondo
5	Niger	Taraba	Kebbi	Imo	Delta	Osun
6	Plateau	Yobe	Sokoto		Edo	Oyo
7	Abuja (FCT)					

### Data Source and Analysis

The temporal data for the national level from 2015 to 2018 of food crop production and the percentage of crops allocated for food, feed, losses, or processed in the country were compiled from the food balance sheets of the Food and Agriculture Organization. In addition, due to the paucity of data at the state level, spatial data indicating the 2017 local food production and harvested area of major crops cultivated across the 36 states in Nigeria grouped in six geopolitical zones were collated from the Federal Ministry of Agriculture and Rural Development (FMARD). The analysis is estimated at two levels, i.e., the national crop yield and percentage of the crop production allocation for food or other purposes from 2015 to 2018 and the state level from local farmers’ food production statistics in 2017.

At the national level, the food production and sufficiency potential were estimated based on a direct relationship between crops harvested area and productivity which gives us the yield per kg/ha/year, and the percentage of crop allocation for food, feed, losses, or processed in the country was estimated from equation (1) below.

The food crops estimated at the national level were rice (*Oryza sativa*), maize (*Zea mays*), sorghum (*Sorghum bicolor*), soya bean (*Glycine max*), cassava (*Manihot esculenta*), and yam (*Dioscorea* spp.). We considered these food crops based on data availability, their ability to provide satiety, and major foods consumed by most households.

The percentage allocation for the highlighted purpose was derived using the following equation:


(1) $$Q_{n}\,\mathrm{crop}\,\mathrm{allocation}\,\mathrm{for}\,{\mathrm{Qn}}_{1}\,\ldots_{5}=100\%\times \frac{Q_{n}}{\Sigma \,\left(f\,o\,o\,d+f\,e\,e\,d+s\,e\,e\,d+l\,o\,s\,s\,e\,s+p\,r\,o\,c\,e\,s\,s\,e\,d\right)}$$

where Q_*n*_ is food crops in our study.

Therefore, we can estimate Qn_1…5_ = crop [*food*_1_, *feed*_2_, *seed*_3_, *losses _4_* and *processed*_5_].

At the state level, the study also compares crop production and food supplied (kg/capita/year) in the 36 states grouped in six geopolitical zones of the country using acquired local food production and population data. The food crops collated at the state level are the same as the ones assessed at the national level but with the removal of soya bean (*Glycine max*), and being replaced with another legume, i.e., cowpea (*Vigna sinensis Savi*) due to data availability at the state level.

## Results

### National Crop Yield and Crop Allocation From Production

**FIGURE 1:**
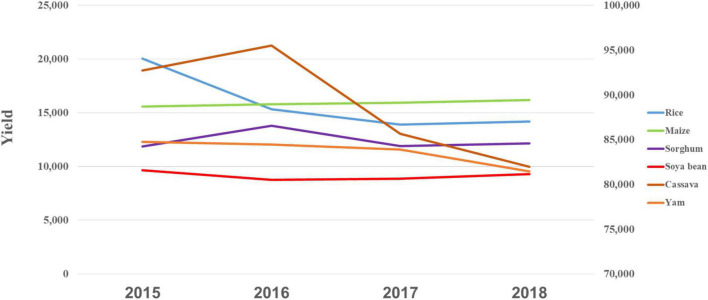
Crop yield from 2015 to 2018 (FAOSTAT).

The results from the national food production indicate the yields per kg/ha/year for rice; cassava and yam decreased from 2015 to 2018, while maize, sorghum, soya bean, and cowpea showed minimal yield increase (Figure 1). However, the yield of these major food crops is low *vis-à-vis* the population demand.

The evaluation of cereal yields showed that rice decreased from 2015 to 2018 despite an increase in the harvested area, while maize had a slight increase in harvested area and a minimally high yield increase from 2015 to 2018. For sorghum, which is a crop mainly cultivated in the North, its harvested area reduced and the yield remained relatively constant, while soya bean had the lowest harvested area, and yield was almost as high as sorghum. In addition, yields from starchy roots also declined from 2015 to 2018, irrespective of an increased harvested area (Appendix Table 1).

In terms of crop allocation from equation (1), the results show that 95.6% of the rice produced was consumed as food, 3.4% was losses, 1% was used as seed, and none was used or processed as livestock feed from 2015 to 2018. Of the maize produced, 54–59% was available as food, 9.3–10% was losses, 0.9–1.2% was reserved as seed, 27.8–32.2% was used as feed, and 2.7–2.9% was processed. About 80% of sorghum production was available for food consumption, 4.5–4.9% was losses, 1.6–1.8% was reserved as seed, 10.5–10.7% were used as feed, and 2.5% was processed. For soya bean, 14.8–18.6% was consumed as food, 6.2–7.1% was losses, 2.4–3.6% was reserved as seeds, 5.6–13.8% was used as feed, and most of the production amounting to 59.2–71% was processed. For cassava, 42.9% was available for consumption as food, 7–8% was losses, and 49.6% was used as feed, and neither for seed nor processed. Finally, for yam, 80% was consumed as food, 12.3–13.4% was losses, 6.9–7.6% was used as feed, and similar to cassava, seeds and processed were not appropriated.

### State-Level Production and Contribution to Food Supply

The results of the local food production in various states across the country in 2017 primarily indicate the cropping pattern and its resulting output. Therefore, it is observed that rice, maize, cowpea, and sorghum are well cultivated and produced in the North, while cassava production thrived in the South, and yams were more produced across the middle belt of the country. In addition, the six geopolitical zones food production further highlighted their food supply potential.

The North Central had a high production of starchy roots and tubers, with the highest production of yams, while maize had the highest production output of cereals, followed by rice, and the least production was cowpea. In the North East, the production of starchy roots and tubers was high, especially yam, while maize production remained high compared to rice, sorghum, and cowpea. The North West produced more cassava and yams, while sorghum production was the highest among cereals, followed by maize and rice.

The Southern zones performed differently in terms of quantities produced. However, the trend of producing more starchy roots and tubers compared to cereals is evident. In the South East states, more cassava is produced than yams. Maize production was high, followed by rice and cowpea. The South South zones produced more cassava and yam, and among cereals, maize production was highest, followed by rice and cowpea. A similar production trend is observed in the South West; cassava and yams are high, while maize is the highest produced cereal, followed by rice and cowpea. Due to paucity of data for sorghum production in the Southern parts of the country, the study recorded no sorghum production for the South East, South South, and South West (Table 2).

**TABLE 2:** 36 states grouped in six geopolitical zones outlook on population, and local production in 2017 (24).

Zones	Population (2016)	Crops cultivated	Production (tonnes/year) 2017
North Central		Rice	3,017,343
		Maize	5,906,673
	29,252,408	Sorghum	1,538,754
		Cowpea	840,834
		Cassava	14,650,656
		Yam	18,535,556
North East	26,263,866	Rice	1,376,408
		Maize	3,145,372
		Sorghum	1,914,410
		Cowpea	1,145,554
		Cassava	3,515,820
		Yam	5,444,699
North West	48,942,307	Rice	1,993,048
		Maize	2,826,998
		Sorghum	3,207,501
		Cowpea	860,536
		Cassava	4,559,313
		Yam	3,380,686
South East	21,955,414	Rice	398,354
		Maize	624,629
		Sorghum	*nil*
		Cowpea	307,062
		Cassava	10,132,258
		Yam	8,743,928
South South	28,829,288	Rice	464,440
		Maize	724,545
		Sorghum	*nil*
		Cowpea	181,772
		Cassava	11,075,538
		Yam	10,023,219
South West	38,257,260	Rice	576,532
		Maize	1,678,330
		Sorghum	*nil*
		Cowpea	538,983
		Cassava	11,135,146
		Yam	7,955,002

**FIGURE 2:**
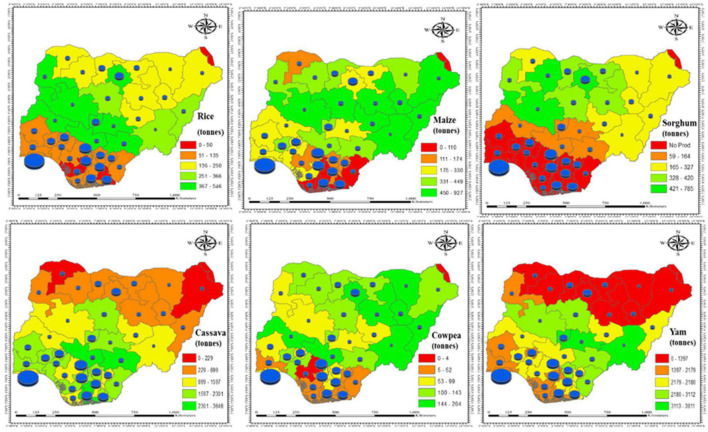
Food production of major staples and population densities across Nigeria in 2017 (24).

A significant observation from the results is that the densely populated states on the production map with low to medium food production are likely to face higher food expenditures due to the forces of demand and supply (Figure 2).

### The Gap in Food Supply at the National and State Levels

**FIGURE 3:**
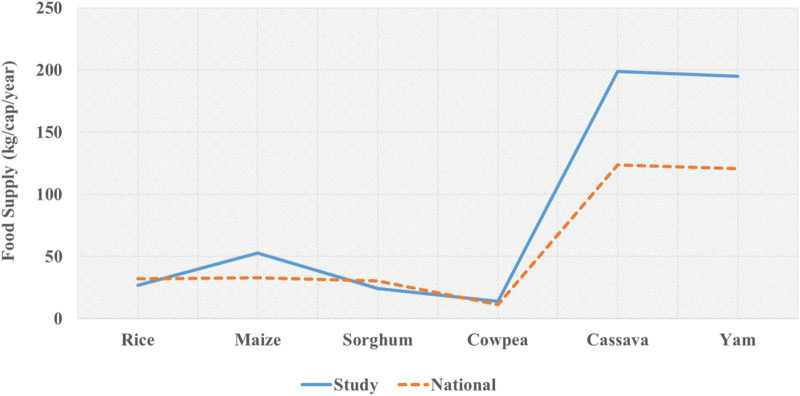
Farmers food supply contribution to the food system in 2017 (FAOSTAT and Study).

To determine the food supply (kg/cap/year) from local production at the state level, an assumed adjustment of 30% was made to account for post-harvest losses, livestock feed, and quantities of food sold on the local market, as no data are available for these indicators at the state level, and the result was divided by the 2016 population data. Therefore, the results from local farmer’s food production statistics show the supply of rice (26.6 kg/cap/year), maize (52.6 kg/cap/year), sorghum (24 kg/cap/year), cowpea (14 kg/cap/year), cassava (198.6 kg/cap/year), and yam (195.1 kg/cap/year). Therefore, the gap between the national and state level per capita food supply (kg/cap/year) are shown in Figure 3.

Generally, considering the aforementioned per capita per year (kg/cap/year) supply, the low per capita consumption is a serious indication of low food production and access in the country. In contrast, the supply gap between the state level (from the study) and the national food supply (as estimated by FAO) elucidates that cassava and yam are substantial to provide per capita satiety, while maize, which is still low can be fairly considered when compared with rice, sorghum, and cowpea because these other food crops have very low per capita consumption. Therefore, the country’s food supply is suboptimal, and food insecurity will be exacerbated if there is no improvement in per capita food supply, considering future population growth. For brevity, in China, the supply of rice in 2017 was 123 kg/cap/year, while the supply in our study was 27 kg/cap/year and the national supply was 32 kg/cap/year in the same year (25). This clearly indicates, cereal production and supply are still low, while starchy roots and tubers are high in the country.

## Discussion

The findings of this study reiterate the cropping and production patterns of major food crops across the country. The national food production showed low and declining yields of rice, sorghum, soya bean, cassava, and yam except for maize from 2015 to 2018, despite an increase in harvested area. The problem of low yield despite farmland expansion was also observed in a study of cereal production in 10 African countries (26). Hence, persistent low yields put the country in a precarious situation of food scarcity in terms of local food supply, leaving thousands of households in Nigeria at a risk of hunger. This is also a major concern considering the Global Hunger Index which ranked Nigeria 98th out of 107 countries in 2020 and is currently 103rd out of 116 countries (27).

From the percentage of crop allocation for food, it can be deduced that despite the percentage of rice available for consumption, it is not enough to meet the demand as results show the per capita consumption is low. About 28–32% of maize produced from 2015 to 2018 is processed into livestock feed, leaving about 60% of maize consumed as food. The growing demand for the fortification and use of maize as animal feed will affect the quantity available for consumption.

Sorghum had 80% of its production available for consumption, which shows it is another major staple in Nigeria, grown more in the North (28). Apart from its nutritional qualities, it is also a component of feed for livestock and in the beverage industry. For instance, aside from its direct consumption, India uses sorghum as livestock feed and as raw material for the alcohol industry (29).

Soya beans are the least directly consumed legumes as 59.2–71% of it is processed into other forms by industry. However, these processed forms are outside the scope of this study, but countries, such as the United States, Brazil, and Argentina, produce biodiesel from soya beans (30).

One of the major staple foods in Nigeria is cassava, 43% of which is available for consumption and 49.6% as feed for livestock. The use of cassava as livestock feed is common in Africa. In Nigeria, it dates back to 1985, when the government banned the importation of maize to be used as a component for livestock feed and mandated alternatives such as cassava (31). Although the ban on maize importation was lifted around the late 1990s, cassava continued to be used in the Nigerian feed milling industry (31). In addition, Nigeria has an estimated 22.5% area under cassava cultivation, which is the largest area under cassava among other countries cultivating cassava in the world (32), and even though cassava is exported; the production is insufficient to meet local demand (33). Therefore, this reiterates the concern noted in this study of population demand outweighing production.

For yams, another important staple, about 80% was available for consumption, but a double-digit percentage was lost during post-harvest.

Overall, this study evaluated the contribution of smallholder farmers to the food supply in 2017, after adjusting for post-harvest losses, livestock feed, and marketed quantities, as compared with the FAO-estimated national food supply for the same year. The study found that rice and sorghum were below FAO national estimates, while smallholder contribution in maize, cowpea, cassava, and yam was higher than FAO national estimates. Thus, smallholder farmers are improving the supply of food, albeit below sufficiency levels.

A significant observation in our study is that food waste due to post-harvest losses remained constant for all the food crops from 2015 to 2018. Post-harvest losses are common in developing countries due to poor storage infrastructure, harvesting techniques, and handling of food crops (34–36).

Of the food crops studied, the North Central region had the highest production, not only in terms of quantity produced, but also in terms of even distribution of production across the major food crops, indicating that the region has favorable climatic conditions for growing these crops. In addition, apart from banditry and insecurity that may have influenced farming activities in the North, the North East, and the North West, cereals and starchy roots had good output. No data on sorghum production were available for the South (i.e. South East, South South, and South West) in this study, which further reduces their potential for food production in this study. Furthermore, the plausible reason for the difference in production in the country can be attributed to the different agroecological conditions, which can influence crop yields (26). The Northern area which experiences sparse rainfall cultivates more cereals, while the Southern area grows more roots and tubers (37), and rainfall increases from South to North. Therefore, the South and North Central zones had more production output from starchy roots and then from maize, rice, and cowpea.

Smallholder farmers’ contribution to food production in Nigeria can be improved to reduce food importation. However, apart from the externalities of climate impacts on food production, smallholder farmers face low support and access to agricultural inputs for their farms. According to the Nigeria Living Standards Measurement Survey of 2019 (45), farmers use inorganic fertilizers (35.4%), organic fertilizers (23.1%), and herbicides (34.7%), and 20.7% participates in extension services (38). To address the challenge of low organic/inorganic fertilizer use, farmers make use of animal waste and compost to enrich the soil. A case study in Ethiopia shows that over 80% of farmers rear livestock for organic manure and as a source of income when sold or rented to be used as farm power (39). However, this method may not be sufficient. Moreover, the Agriculture Orientation Index (AOI) for government expenditure, an indicator that measures government commitment and investment through enhanced international cooperation, rural infrastructure, agricultural research and extension services, technological development, and crop and livestock gene banks to increase agricultural production capacity (40), indicates that Nigeria’s investment in agriculture is inadequate (Appendix 1). This low investment in agriculture is not peculiar to Nigeria but is true of most African countries despite their pledge to promote food security and poverty reduction by signing the Maputo Declaration on Agriculture and Food Security in 2003 (41). In addition, the land tenure system in Nigeria in addition to bureaucracy restrains citizens to acquire lands easily compared to other African countries such as Rwanda and Botswana (42). This limits the ability to improve food production, as demand will be at the mercy of food imports.

## Conclusion

In this study, an attempt was made to ascertain the percentage of crops produced in Nigeria for food or other purposes, and whether smallholder farmers are able to put the country on the path of self-sufficiency and provide satiety from their local production considering their fragmented farm sizes.

In sum, post-harvest losses have been constant from 2015 to 2018 and the percentage allocated to feeding livestock is increasing. At the same time, per capita supply is low for cereals, raising serious concerns about food availability for the teeming population. In order to reduce post-harvest losses and increase food availability, investment in adequate storage facilities for agro-produce is a welcome development as it will not only increase food availability but also improve farmers’ income and livelihoods.

The study showed that smallholder farmers improved food supply; however, it is not able to provide the country with the much-needed production to be self-sufficient, especially in cereals as findings show cereal productions are low and not sufficient. In addition, yields are equally low and declining despite farmland expansion, which poses a food insecurity challenge for a growing population such as Nigeria.

The North Central region shows a promising potential to have the capacity to produce food. However, when given adequate agricultural support, the six geopolitical zones are able to diversify their food productions to crops that are within the agroclimatic conditions, especially cereals, and complement each other.

This study suggests that closing the yield gap, especially for cereals, as an increase in cereal production contributes significantly to an increase in calorie availability and income for farmers and households in the country (18). Furthermore, the study suggests a sustainable balance between the uses of food crops for animal feed to reduce pressure on land resource use. This suggestion aligns with the opinion of available research to reduce the quantity of food crops used for livestock feed, as it is an unsustainable way to provide calories for human consumption and seek alternative ways (10, 43).

In addition, the study suggests private investment and collaborations with international community to improve agriculture and harness the potential of the different agroecological conditions across the country. In the meantime, promoting dry season cultivation through irrigation to encourage dual-season harvest will assist in food production. The country has low irrigated cultivable land and most of the existing irrigation schemes have become obsolete due to high operating costs and poor maintenance culture (44). Therefore, a revamp and adequate funding of critical sectors in charge of water and irrigation access is a step in the right direction. This is because available literature on the use of irrigation has proven to increase crop yield. For example, China and India have the most irrigated areas among developing countries, which ultimately increase their crop yields (3).

Other proposed measures to increase production and food sufficiency are implementing a “crop structural adjustment” program, whereby smallholder farmers in different states are supported and funded by national, state, and nongovernmental agencies to produce crops suited to their different agroclimatic conditions.

Overall, this study provides a useful approach for evaluating local farmer’s contribution to food sufficiency and the need for targeted external support on food production, especially cereals, and the exigency to reduce post-harvest losses.

There are limitations in our study such as the food crops were limited which influenced the outcome of some results particularly in states in the Southern zones of Nigeria such as no sorghum cultivation data in the South. In addition, the FAO food balance sheet had its limitations such as no national data for cowpea, therefore, the reliance on secondary data for the assessment.

There was a paucity of data to show post-harvest losses and the amount of food produced sold or given to livestock at the local farm level and an assumed percentage value was used. This may have influenced the outcome of the study.

In the future, the study serves as a guide for further research and sustainable policy development in this area.

## Data Availability Statement

The original contributions presented in this study are included in the article/Supplementary Material, further inquiries can be directed to the corresponding author/s.

## Author Contributions

JC: conceptualization, investigation, methodology, data, software, writing—original draft, review and editing. LZ: writing—review and editing, supervision, validation, funding acquisition, project administration, and resources. HY, YX, and FM: visualization and writing—review and editing. TL: software, review and editing. All authors have read and approved the final manuscript.

## Conflict of Interest

The authors declare that the research was conducted in the absence of any commercial or financial relationships that could be construed as a potential conflict of interest.

## Publisher’s Note

All claims expressed in this article are solely those of the authors and do not necessarily represent those of their affiliated organizations, or those of the publisher, the editors and the reviewers. Any product that may be evaluated in this article, or claim that may be made by its manufacturer, is not guaranteed or endorsed by the publisher.

## Figures and Tables

**APPENDIX A1:**
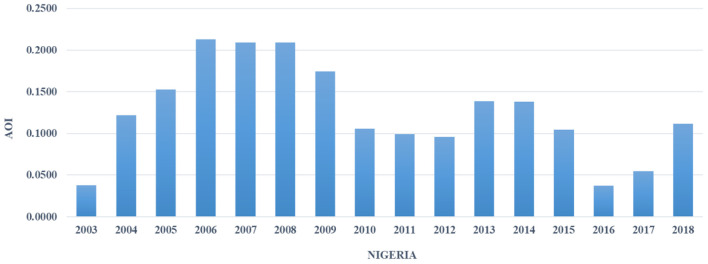
Agriculture orientation index for government expenditure in Nigeria. An AOI > 1 means the agriculture sector receives a higher share of government spending relative to its economic value. An AOI < 1 reflects a lower orientation to agriculture (40).

## Appendix

**TABLE A1:** National harvested area (ha) and yield (kg/ha) (FAOSTAT).

Year	Rice ha 10^5^	Rice Yield (kg/ha)	Maize ha 10^5^	Maize Yield (kg/ha)	Sorghum ha 10^5^	Sorghum Yield (kg/ha)	Soyabean ha 10^5^	Soyabean Yield (kg/ha)	Cassava ha 10^5^	Cassava Yield (kg/ha)	Yam ha 10^5^	Yam Yield (kg/ha)
2015	312	20,042	677	15,599	590	11,875	61	9,658	622	92,727	539	84,748
2016	494	15,326	731	15,793	547	13,809	107	8,751	623	95,545	608	84,475
2017	563	13,906	654	15,933	582	11,923	112	8,877	632	87,138	645	83,900
2018	587	14,306	682	16,138	560	12,151	70	9,334	667	83,679	607	82,402
Graph												

## References

[1] Dessy S Jacques E Isabelle O. *Understanding the Persistent Low Performance of African Agriculture.* Montreal, QC: Research Center on Risk, Economic Stakes and Public Policy (2006). 10.2139/ssrn.905291

[2] Staatz JM Dembele NN. *Agriculture for Development in Sub-Saharan Africa*. Background Paper for the World Development Report 2008. Michigan State University: John M. Staatz and Niama Nango Dembélé (2008).

[3] Alexandratos N Bruinsma J. *World Agriculture Towards 2030/2050: The 2012 Revision*. ESA Working Paper No. 12-03. FAO: Rome. (2012).

[4] Reisch L Ulrike E Sylvia L. Sustainable food consumption: an overview of contemporary issues and policies. *Sustainability.* (2013) 9:7–25. 10.1080/15487733.2013.11908111

[5] Dankumo A Riti JS Ayeni BS. Contribution of agricultural and industrial sectors to the development of Nigerian economy from 1995 to 2012. *Int J Bus Manag Allied Sci.* (2015) 2:2128–35.

[6] Rasul G. Twin challenges of COVID-19 pandemic and climate change for agriculture and food security in South Asia. *Environ Chall.* (2021) 2:100027. 10.1016/j.envc.2021.100027

[7] Lambin EF Turner BL Geist HJ Agbola SB Angelsen A Bruce JW The causes of land-use and land-cover change: moving beyond the myths. *Glob Environ Change.* (2001) 11:261–9. 10.1016/S0959-3780(01)00007-3

[8] Ellis EC Ramankutty N. Putting people in the map: anthropogenic biomes of the world. *Front Ecol Environ.* (2008) 6:439–47. 10.1890/070062

[9] Tilman D Balzer C Hill J Befort BL. Global food demand and the sustainable intensification of agriculture. *Proc Natl Acad Sci U.S.A.* (2011) 108:20260. 10.1073/pnas.1116437108 22106295 PMC3250154

[10] Cassidy ES West PC Gerber JS Foley JA. Redefining agricultural yields: from tonnes to people nourished per hectare. *Environ Res Lett.* (2013) 8:034015. 10.1088/1748-9326/8/3/034015

[11] Foley JA Ramankutty N Brauman KA Cassidy ES Gerber JS Johnston M Solutions for a cultivated planet. *Nature.* (2011) 478:337–42. 10.1038/nature10452 21993620

[12] UNCTAD. Commodities and development report 2015: smallholder farmers and sustainable commodity development. In: *Proceedings of the United Nations Conference on Trade and Development.* Geneva: UNCTAD (2015).

[13] Omotilewa OJ Jayne TS Muyanga M Aromolaran AB Liverpool-Tasie LSO Awokuse T. A revisit of farm size and productivity: empirical evidence from a wide range of farm sizes in Nigeria. *World Dev.* (2021) 146:105592. 10.1016/j.worlddev.2021.105592 34602709 PMC8350315

[14] Chiaka JC Zhen L. Land use, environmental, and food consumption patterns in Sub-Saharan Africa, 2000–2015: a review. *Sustainability.* (2021) 13:8200. 10.3390/su13158200

[15] Le Gal PY Andrieu N Bruelle G Dugué P Monteil C Moulin CH Modelling mixed crop-livestock farms for supporting farmers’ strategic reflections: the CLIFS approach. *Comput Electron Agric.* (2022) 192:106570. 10.1016/j.compag.2021.106570

[16] Fan S-G Chan-Kang C. Is small beautiful? Farm size, productivity, and poverty in Asian agriculture. *Agric Econ.* (2005) 32:135–46. 10.1111/j.0169-5150.2004.00019.x

[17] Muyanga M Jayne TS. Revisiting the farm size-productivity relationship based on a relatively wide range of farm sizes: evidence from Kenya. *Am J Agric Econ.* (2019) 101:1140–63. 10.1093/ajae/aaz003

[18] Chibwana C Fisher M Shively G. Cropland allocation effects of agricultural input subsidies in Malawi. *World Dev.* (2012) 40:124–33. 10.1016/j.worlddev.2011.04.022

[19] Idris-Adeniyi KM Akinbile LA Busari AO Adebooye OC. Factors influencing household food security among MicroVeg project beneficiaries in Nigeria. *Acta Hortic.* (2019) 1238:105–16. 10.17660/ActaHortic.2019.1238.12

[20] Ricciardi V Ramankutty N Mehrabi Z Jarvis L Chookolingo B. How much of the world’s food do smallholders produce? *Glob Food Secur.* (2018) 17:64–72. 10.1016/j.gfs.2018.05.002 30229072

[21] FAO. *Food and Agricultural Organization of the United Nations Statistics Annual Population.* Rome: FAO (2018).

[22] Matemilola S. The challenges of food security in Nigeria. *Open Access Libr J.* (2017) 4:1. 10.4236/oalib.1104185

[23] Adewole OO Serifat F. Modelling rainfall series in the geo-political zones of Nigeria. *J Environ Earth Sci.* (2015) 5:100–11.

[24] FMARD. *Federal Ministry of Agriculture and Rural Development, Nigeria* Abuja: FMARD (2017). Available online at: https://fmard.gov.ng/

[25] FAOSTAT. *Crops and Livestock Products. Food and Agriculture Organization of the United Nations.* Rome: FAOSTAT (2022).

[26] Ittersum MKV Bussel LGJV Wolf J Grassini P Wart JV Guilpart N Can Sub-Saharan Africa feed itself? *Proc Natl Acad Sci U.S.A.* (2016) 113:14964–9. 10.1073/pnas.1610359113 27956604 PMC5206509

[27] GHI. *Global Hunger Index – Global Hunger by Severity. Concern Worldwide and Welthungerhilfe.* New York, NY: GHI (2021).

[28] Chukwu AJ Agbagwa IO. Phytogeographic distribution of *Sorghum* in Nigeria. *Int J Sci Res Publ.* (2020) 10:232–6. 10.29322/IJSRP.10.01.2020.p9736

[29] Gali B Rao PP. Regional analysis of household consumption of *Sorghum* in major *Sorghum*-producing and *Sorghum*-consuming states in India. *Food Secur.* (2012) 4:209–17. 10.1007/s12571-012-0189-9

[30] Knoope MMJ Balzer CH Worrell E. Analysing the water and greenhouse gas effects of soya bean−based biodiesel in five different regions. *GCB Bioenergy.* (2019) 11:381–99. 10.1111/gcbb.12558

[31] Tewe OO. *The Global Cassava Development Strategy – Cassava for Livestock Feed in Sub-Saharan Africa.* Rome: Food and agriculture organization of the United Nations (2004).

[32] Obayelu OA Ebute S. Assessment of cassava supply response in Nigeria using vector error correction model (VECM). *Agricultura.* (2016) 13:79–86. 10.1515/agricultura-2017-0010

[33] Ikuemonisan ES Mafimisebi TE Ajibefun I Adenegan K. Cassava production in Nigeria: trends, instability and decomposition analysis (1970 2018). *Heliyon.* (2020) 6:e05089. 10.1016/j.heliyon.2020.e05089 33072906 PMC7553005

[34] Prusky D. Reduction of the incidence of postharvest quality losses, and future prospects. *Food Secur.* (2011) 3:463–74. 10.1007/s12571-011-0147-y

[35] Affognon H Mutungi C Sanginga P Borgemeister C. Unpacking postharvest losses in Sub-Saharan Africa: a meta-analysis. *World Dev.* (2015) 66:49–68. 10.1016/j.worlddev.2014.08.002

[36] Chegere MJ. Post-harvest losses reduction by small-scale maize farmers: the role of handling practices. *Food Policy.* (2018) 77:103–15. 10.1016/j.foodpol.2018.05.001

[37] Chapin Metz H. *Nigeria: A Country Study.* Washington, DC: GPO for the Library of Congress (1991).

[38] NBS. *General Household Survey in Nigeria (Living Standard Measurement Survey Wave 4).* Nigeria: National Bureau of Statistics (2019).

[39] Haji J. Production efficiency of smallholders’ vegetable-dominated mixed farming system in eastern Ethiopia: a non-parametric approach. *J Afr Econ.* (2007) 16:1–27. 10.1093/jae/ejl044 28159496

[40] FAO. *Agriculture Orientation Index for Government Expenditure in the World (2001 – 2019).* Rome: FAO (2021).

[41] AU. *“Maputo Declaration on Agriculture and Food Security” – Assembly of the African Union.* Maputo: Africa Union (2003).

[42] Udoekanem NB Adoga DO Onwumere VO. Land ownership in Nigeria: historical development, current issues and future expectations. *J Environ Earth Sci.* (2014) 4:182–9.

[43] Pinotti L Luciano A Ottoboni M Manoni M Ferrari L Marchis D Recycling food leftovers in feed as opportunity to increase the sustainability of livestock production. *J Clean Prod.* (2021) 294:126290. 10.1016/j.jclepro.2021.126290

[44] Adelodun B Choi K-S. A review of the evaluation of irrigation practice in Nigeria: past, present and future prospects. *Afr J Agric Res.* (2018) 13:2087–97. 10.5897/AJAR2018.13403

[45] NBS Nigeria Bureau of Statistics. *LSMS-Integrated Surveys on Agriculture General Household Survey Panel.* Nigeria: National Bureau of Statistics (2019).

